# Regulation of miRNAs by Natural Antioxidants in Cardiovascular Diseases: Focus on SIRT1 and eNOS

**DOI:** 10.3390/antiox10030377

**Published:** 2021-03-03

**Authors:** Yunna Lee, Eunok Im

**Affiliations:** College of Pharmacy, Pusan National University, Busan 46241, Korea; yunnalee@pusan.ac.kr

**Keywords:** natural antioxidants, miRNAs, eNOS, SIRT1, endothelial dysfunction

## Abstract

Cardiovascular diseases (CVDs) are the most common cause of morbidity and mortality worldwide. The potential benefits of natural antioxidants derived from supplemental nutrients against CVDs are well known. Remarkably, natural antioxidants exert cardioprotective effects by reducing oxidative stress, increasing vasodilation, and normalizing endothelial dysfunction. Recently, considerable evidence has highlighted an important role played by the synergistic interaction between endothelial nitric oxide synthase (eNOS) and sirtuin 1 (SIRT1) in the maintenance of endothelial function. To provide a new perspective on the role of natural antioxidants against CVDs, we focused on microRNAs (miRNAs), which are important posttranscriptional modulators in human diseases. Several miRNAs are regulated via the consumption of natural antioxidants and are related to the regulation of oxidative stress by targeting eNOS and/or SIRT1. In this review, we have discussed the specific molecular regulation of eNOS/SIRT1-related endothelial dysfunction and its contribution to CVD pathologies; furthermore, we selected nine different miRNAs that target the expression of eNOS and SIRT1 in CVDs. Additionally, we have summarized the alteration of miRNA expression and regulation of activities of miRNA through natural antioxidant consumption.

## 1. Introduction

### 1.1. Oxidative Stress and Endothelial Dysfunction in Cardiovascular Diseases (CVDs)

CVDs are the most common cause of morbidity and mortality worldwide, accounting for an estimated 31.5% of the 54 million total global deaths reported in 2013 [1]. CVDs are multifactorial disorders that affect the heart and blood vessels and include coronary artery disease (CAD), stroke, hypertension, atherosclerosis, atrial fibrillation, myocardial infarction (MI), and ischemic heart failure [2]. The main risk factors for CVD-associated mortality are high blood pressure, smoking, diabetes mellitus, obesity, and aging [1]. These risk factors are highly associated with the overproduction of reactive oxygen species (ROS) and the accumulation of oxidative damage [3]. ROS is a collective term that includes both oxygen-derived free radicals, such as superoxide anions (O_2_^•−^), hydroxyl radicals (^•^OH), and nitric oxide (^•^NO) with unpaired electrons in their outer orbital, and other pro-oxidant and non-radical oxygen derivatives that are easily converted into radicals, such as hydrogen peroxide (H_2_O_2_) and peroxynitrite (ONOO^−^) [4,5].

ROS production is an unavoidable process of aerobic life in living cells and demonstrates beneficial functions in host defense [4]. ROS show increased interactions with small inorganic molecules, proteins, lipids, carbohydrates, and nucleic acids, and alter the function of these target molecules [6]. Physiological ROS play a protective role against cellular damage inflicted by pathogens, play an important role in intracellular signaling as second messengers, and regulate a considerable number of regulatory processes in all cells and tissues [6]. In healthy conditions, the burden of ROS is regulated by the action of enzymatic and non-enzymatic antioxidant systems that scavenge and neutralize free radicals [7]. However, redox imbalance caused by excessive ROS production and/or insufficient scavenging mechanisms results in oxidative stress, which causes the dysregulation of many biological processes involved in the pathogenesis of CVDs [4]. Increased ROS production has already been reported in several cardiac diseases. Plasma nitrite, NO synthase (NOS), and superoxide production increase markedly in left ventricular (LV) cardiac hypertrophy and chronic heart failure (CHF) in vivo [8,9]. Moreover, scavenging ROS by overexpressing catalase (CAT) ameliorates LV cardiac hypertrophy, fibrosis, and diastolic dysfunction, and attenuates the phenotype of heart failure [10,11].

Increased oxidative stress is known to be associated with the impairment of vascular endothelial function, and then deleterious alterations of endothelial physiology are involved in the development of CVDs [2]. The vascular endothelium is a multifunctional organ and is critically involved in the modulation of vascular tone and structure for maintenance of vascular health [12]. This endothelial cell monolayer coating the vascular lumen is a mechanical and biological barrier between the blood components and the vascular wall [3]. Endothelial dysfunction indicates impaired endothelium-dependent vasodilation, is the common pathological basis of CVDs, and is associated with high incidence, morbidity, and mortality rates [13]. It is a key early step in the development of atherosclerosis and is involved in plaque progression [3]. Moreover, endothelial dysfunction can increase the risk of atherosclerosis development even in a normotensive and symptom-free condition in children and young adults [14]. Impairment of vasodilation and endothelial dysfunction were also associated with the development of CAD [15]. Therefore, a better understanding of the mediators involved in the development of endothelial dysfunction-induced CVDs is necessary to obtain valuable targets for preventing the development of CVDs. Studies on the expression of microRNAs (miRNAs) under oxidative stress provides therapeutic clues for the treatment of the dysfunction of the vascular system and for elucidation of the pathology of CVDs.

### 1.2. Natural Antioxidants in Cardioprotection 

**Figure 1:**
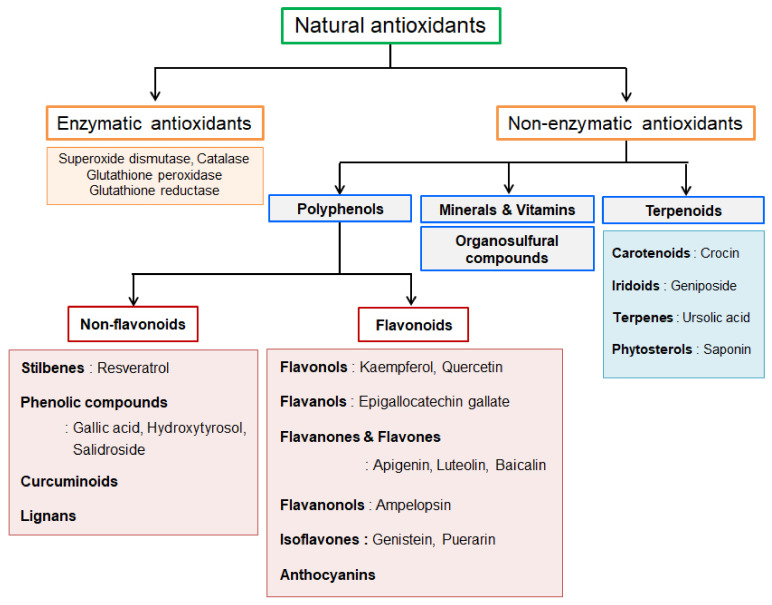
Classification of natural antioxidants. Natural antioxidants are categorized into enzymatic and nonenzymatic groups. Enzymatic antioxidants are endogenously produced in our body, whereas nonenzymatic antioxidants are constituents of many fruits and vegetables. One of the largest class of dietary antioxidants polyphenols includes the non-flavonoid subgroup and the flavonoid subgroup.

Natural antioxidants include synthesized molecules in the human body produced via metabolic processes and obtained through supplemented nutrition from other natural sources. These natural sources exert health-promoting effects via their phytochemical contents, which possess antioxidant abilities [16]. Natural antioxidants can be classified into two major groups, namely enzymatic and nonenzymatic (Figure 1). Enzymatic antioxidants are produced endogenously, such as superoxide dismutase (SOD), CAT, glutathione peroxidase, and glutathione reductase [17]. These enzymes contribute to the total antioxidant capacity of the body. In contrast, nonenzymatic antioxidants are obtained from dietary sources, including polyphenols, terpenoids, organosulfur compounds, vitamins, and minerals such as zinc and selenium [18]. Nonenzymatic antioxidants are widely distributed in food, fruits, vegetables, tea, and traditional medicinal herbs [19].

Polyphenol compounds, constituting the largest class of dietary antioxidants, are secondary metabolites of plants and can be further classified into two main groups, namely flavonoids and non-flavonoids, according to their chemical structure and orientation of the number of phenol rings bound to one another [20]. The basic structure of non-flavonoid compounds contains a single aromatic ring, including phenolic compounds, stilbenes, curcuminoids, and lignans [20]. In contrast, all flavonoids share the basic structure of two or more aromatic rings connected to three carbons [20]. Flavonoids are the most abundant polyphenols and account for 60% of the human diet [21]. The structural diversity of flavonoids is acquired by variations in hydroxylation and oxidation states; flavonoids are subdivided into different subgroups including flavones, flavonols, flavanols, flavanones, flavanonols, isoflavones, and anthocyanins [20]. 

The beneficial effects of these dietary antioxidants, especially polyphenols, against CVDs have been extensively investigated throughout the years [22]. The long-term consumption of polyphenol-rich diets plays a vital role in producing cardiovascular benefits through an improvement in vascular function and modulation of inflammation, suggesting considerable potential for the future management of CVDs [23]. Fruits are the richest source of phenolic compounds, and in one study, total fruit intake correlated with improved cardiovascular health, showing a 6%–7% reduction in the risk of CVD mortality [24]. Phenolic compounds in all fruits and vegetables confer protection against CVDs by reducing cholesterol, inflammatory molecules, and arterial stiffness, and by increasing the antioxidant capacity [25]. 

Although nutritional supplements primarily reduce morbidity and mortality via exertion of cardio-protective effects, several studies have attempted to elucidate the mechanisms underlying oxidative stress; however, the specific molecular mechanisms of these dietary interventions have not been extensively studied. Increasing evidence has revealed that modulation of miRNA expression via the action of dietary compounds activates their potential capacity to mitigate the pathology of CVDs. Dietary polyphenols affect epigenetic events, posttranslational modifications, and miRNA expression, suggesting potential abilities to be considered for the development of drugs for therapeutic intervention [26]. Additionally, bioactive compounds present in foods may influence endogenous miRNA biogenesis [27]. Hence, targeting miRNAs using polyphenols will be a promising therapeutic strategy against CVDs.

### 1.3. Biogenesis and Functions of microRNA (miRNA)

**Figure 2:**
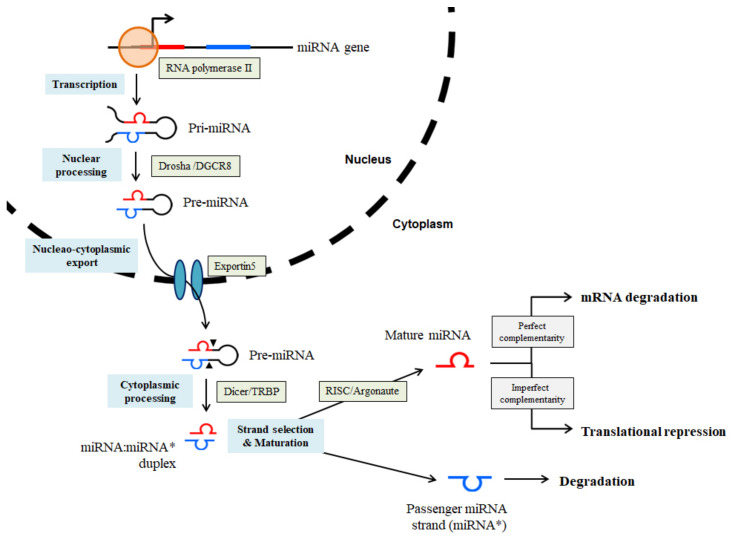
microRNA (miRNA) biogenesis. Biogenesis of miRNA begins with the generation of the pri-miRNA transcript. The microprocessor complex, Drosha/DGCR8, produces the pre-miRNA by cleaving pri-miRNA. The pre-miRNA is exported to the cytoplasm in an Exportin 5-dependent manner and undergoes cytoplasmic processing to produce the mature miRNA duplex (miRNA:miRNA*, passenger strand indicated by an asterisk) by the Dicer/ transactivation response element RNA-binding protein (TRBP) complex. Either the miRNA or miRNA* strands of the mature miRNA duplex is assembled into the effector complex RNA-induced silencing complex (RISC). The passenger miRNA strand is degraded and mature miRNA functions as a guide by base-pairing with the target mRNA to negatively regulate its expression. The direct gene silencing induced by mature miRNA via mRNA cleavage or translation repression is based on the level of complementarity between the miRNA and the mRNA target.

A miRNA is a small non-coding RNA molecule containing 21–24 nucleotides, which can regulate gene expression by binding to the three prime untranslated regions (3’-UTRs) of target genes. Generally, miRNAs act as negative regulators of gene expression by inducing RNA silencing or translational repression [28]. miRNA genes are located within the introns and exons of protein-coding mRNAs or expressed as independent transcripts containing multiple miRNAs [4]. Biogenesis of miRNA begins with the activity of RNA polymerase II, whose action forms a long primary transcript (pri-miRNA) from an imperfect ~80 nt RNA hairpin. The pri-miRNA contains the active miRNA in a stem-loop structure. This upper hairpin of pri-miRNA is cleaved by Drosha RNase III, complexed to the RNA-binding protein DGCR8, to produce a ~65-nt short hairpin intermediate (precursor, pre-miRNAs). The pre-miRNA is transported to the cytoplasm by the nuclear export factor Exportin 5, and further processed by Dicer RNase III, complexed to TAR RNA-binding protein, to form ~20-nt miRNA:miRNA* double-stranded RNA molecules. The complementary strand (miRNA*) is degraded, whereas another strand (miRNA) of this duplex forms the mature miRNA, which is incorporated into the RNA-induced silencing complex (RISC) together with the Argonaute proteins. RISC recognizes the complementary mRNA transcript and binds to the 3′-UTR of the target mRNA. The complete pairing of miRNA-mRNA to the first 8 nt within the 5′ miRNA end results in the formation of the seed sequence. This complimentary seed pairing of mRNA/miRNA leads to the degradation of the target mRNA or translational inhibition of functional proteins [4,29,30,31]. Generally, high miRNA-target complementarity is necessary for mRNA degradation, whereas the translational repression is characterized by low miRNA-target complementarity [32] (Figure 2). Each miRNA has hundreds of target mRNAs, while a single mRNA is the target of multiple miRNAs, thus implying that miRNAs tightly control the physiology of mammals [33]. Target mRNA regulation by miRNAs is extremely complex and remains unknown; however, the importance of fine-tuning of the modulation performed by miRNAs in biological mechanisms is evident through various reports [4]. Abnormal changes in miRNA expression have been closely linked with the onset and progression of many pathological conditions. They have recently gained popularity as important posttranscriptional modulators of various biological processes such as proliferation, differentiation, apoptosis, stress resistance, and angiogenesis [34,35]. 

Several miRNAs are abundant in the vascular system and are involved in the regulation of endothelial function [28]. There is a growing interest in the development of miRNA-targeted therapies for vascular diseases because miRNAs also play crucial roles in the maintenance of cardiovascular tissue homeostasis [29]. The first correlation between miRNA expression and heart disease development was revealed in 2006 [30]. Since then, many follow-up studies have unraveled the critical role played by miRNAs in the pathophysiology of the cardiovascular system [31,32]. The alteration of miRNA expression and gain- and loss-of-function of miRNAs have been demonstrated in the development of cardiovascular disorders, including cardiac hypertrophy, cardiac fibrosis, cardiomyopathy, aortic stenosis, myocardial infarction, coronary heart disease, and atherosclerosis [33,34,35,36,37]. In this review, we have discussed nine miRNAs whose expression levels are highly related to the regulation of cardiovascular function. miR-155 is a novel component of inflammatory signal transduction in atherosclerosis and is considered an athero-miRNA [38]. The expression of the members of the miR-15 family, including miR-15 and miR-16, is upregulated in several heart diseases, including ischemia-reperfusion injury, cardiac hypertrophy, and heart failure [4,39]. miR-221 and its paralogue miR-222 are highly expressed in endothelial cells and show notable activities in the vascular network by influencing the angiogenic properties of endothelial cells [28,40]. miR-210 is associated with angiogenesis and enhances microcirculation under both physiological and pathophysiological conditions [41,42]. miR-126 is considered a pivotal endothelial-specific miRNA, one of the most abundantly expressed in vascularized tissues and endothelial cells, and is involved in cardiac angiogenesis [12,42]. Alteration of the miR-21, miR-199a, miR-34a, and miR-145 expression levels has also been linked to a variety of cardiac injuries, with the levels showing increases in the affected cardiac tissue [43,44,45,46]. This review will provide new insights into the importance of these nine miRNAs in the regulation of oxidative stress in cardiovascular pathologies and the miRNA-related molecular mechanisms of the beneficial effects of natural antioxidants in the treatment of heart diseases.

## 2. Endothelial Nitric Oxide Synthase (eNOS) and Sirtuin 1 (SIRT1) Interaction in Endothelial Dysfunction

### 2.1. The Roles of eNOS in Endothelial Dysfunction

**Figure 3:**
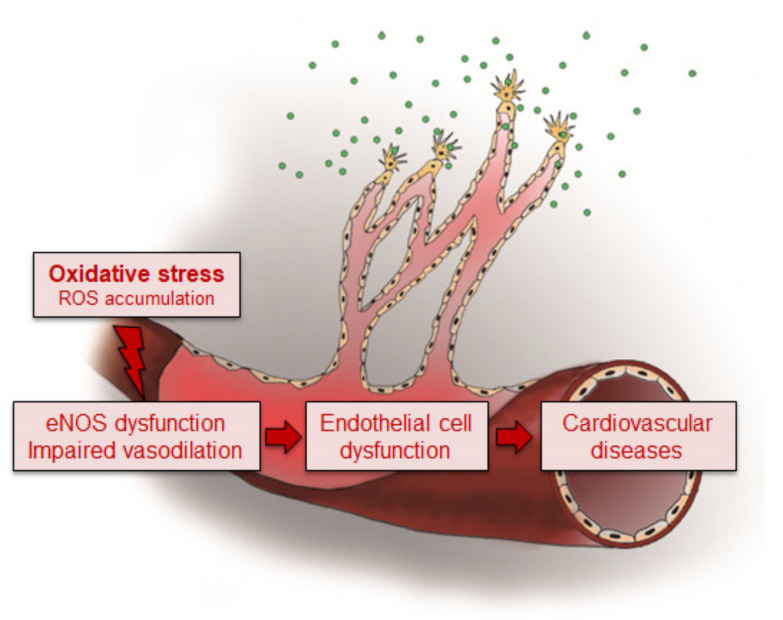
Oxidative stress induces the progression of cardiovascular diseases (CVDs). Oxidative stress plays a central role in the pathogenesis of CVDs. Excessive reactive oxygen species (ROS) causes damage to the cellular structure in the vascular wall and induces endothelial nitric oxide synthase (eNOS) dysfunction, leading to the impairment of vasodilation by the reduction in nitric oxide (NO) bioavailability. These changes contribute to the structural and functional alteration of the vasculature. The endothelial dysfunction is highly associated with the development of CVDs as an initial step in the process of pathogenesis.

Under oxidative stress, ROS induces endothelial dysfunction by disrupting the vasoprotective NO signaling pathway, leading to NOS uncoupling [2]. There are three types of NOS, namely type I neuronal NOS (nNOS), type II inducible NOS (iNOS), and type III endothelial NOS (eNOS) [47]. Among them, eNOS is mainly expressed in endothelial cells and is also detected in cardiomyocytes, platelets, hippocampal neurons, and epithelial cells [48]. Expression of eNOS in endothelial cells is markedly reduced in hypertensive rats compared with age-matched normotensive rats [49]. However, endothelial dysfunction is associated with increased eNOS expression rather than its reduction, because the functional activity is more important than the change in expression of eNOS in the determination of NO bioavailability. Dysfunction of eNOS accelerates atherosclerotic lesion formation in mice [50]. Under conditions of endothelial dysfunction, the upregulation of eNOS expression is induced by elevated production of H_2_O_2_, which decreases the bioactivity of NO, resulting in the exhibition of beneficial actions in disease states [51]. Therefore, endothelial dysfunction with reduced NO bioavailability and increased oxidant excess contributes to the initiation and progression of atherosclerotic plaque formation, consequently triggering cardiovascular events [52] (Figure 3).

**Figure 4:**
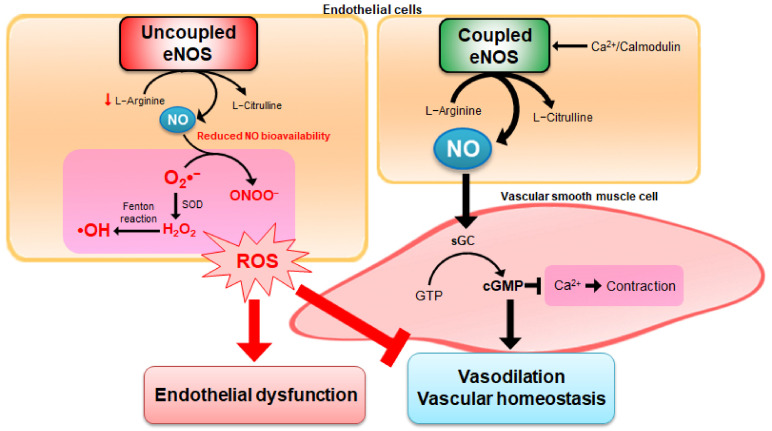
Endothelial dysfunction via modulation of eNOS uncoupling. Coupled eNOS utilizes _L_-arginine to produce NO and _L_-citrulline predominantly rather than ROS. NO plays an important role in the vasodilation of vascular smooth muscle cells (VSMCs) to maintain vascular homeostasis through the cyclic guanosine monophosphate (cGMP)-dependent signaling cascade. In pathological situations, eNOS becomes dysfunctional and produces superoxide anions (O^2•−^) rather than NO. Superoxide anions are dismutated to form hydrogen peroxide (H_2_O_2_) by superoxide dismutase (SOD), and are used to generate hydroxyl radicals (^•^OH) by the interaction with ferrous iron and H_2_O_2_ through Fenton reaction. Furthermore, the interaction between NO and superoxide anions leads to the formation of peroxynitrite (ONOO^−^), resulting in ROS production with subsequent endothelial dysfunction. Endothelial dysfunction is characterized by decreasing bioavailability of eNOS-derived NO, which results in impaired endothelium-dependent vasodilation.

Ca^2+^-activated calmodulin is important for eNOS activity. Ca^2+^ entry into endothelial cells activates the calmodulin-binding domain of eNOS [47]. This stimulation transforms _L_-arginine into _L_-citrulline, leading to NO production. Synthesized NO diffuses from the endothelial cells to the vascular smooth muscle cells (VSMCs), stimulates the soluble guanylate cyclase (sGC), and induces the formation of cyclic guanosine monophosphate (cGMP). VSMCs are predominant constituents of blood vessels and dynamic components produced in response to vasoactive stimuli [4]. Increasing cGMP levels in VSMCs decreases the intracellular concentration of Ca^2+^, causes relaxation of vascular smooth muscle, and, thus, acts as a potent vasodilator [53]. Under several pathological conditions, active eNOS formation with oxygenase activity and NO production is severely reduced, leading to uncoupling of eNOS. Dysfunctional eNOS activates the reductase function of eNOS, and induces more ROS formation by switching from NO to superoxide anions production and ONOO^−^, consequently reducing the bioavailability of NO and vasoconstriction [2]. Dioxygen (O_2_) is involved in the initial step for ROS formation, and O_2_ results in the production of superoxide anions by electron capture [2]. Superoxide anions can interact with NO and affect the abnormality of endothelial functions. Superoxide anions can be converted to H_2_O_2_ by the action of SOD enzymes, and generate hydroxyl radicals by Fenton reaction between ferrous iron and H_2_O_2_ interaction [54,55] (Figure 4). Consequently, the bioavailability of NO can be decreased by: (1) reduction in eNOS expression or activity, (2) eNOS uncoupling to produce superoxide anions, and (3) NO degradation by reacting with superoxide anions, leading to the formation of ONOO^−^ [56]. As NO plays a key role in the physiological regulation of the cardiovascular system, abnormalities in the bioavailability of NO accompany the pathologies observed in hypertension, atherosclerosis, and angiogenesis-associated disorders [47]. 

### 2.2. Interaction between SIRT1 and eNOS

SIRT1 is the most extensively studied member of the highly conserved sirtuin family and acts as a nicotinamide adenine dinucleotide (NAD)-dependent histone deacetylase, which regulates various biological processes including oxidative stress, metabolism, and aging [57]. SIRT1 is ubiquitously expressed in the vasculature, including endothelial cells, smooth muscle cells, and perivascular adipose tissues [58]. A growing number of studies have focused on the role played by SIRT1 in endothelial cell biology because of its antioxidative and anti-inflammatory properties [59,60]. Endothelial cell-specific deletion of SIRT1 in mice impaired the formation of new vessels in response to angiogenic cues [60]. The reduced expression of SIRT1 is consistent with the increased levels of acetylation found in heart failure [61]. Overexpression of SIRT1 exhibits protective effects against HF, including increased cell viability, reduced apoptosis, and improved heart function in a rat model [62]. These beneficial effects of SIRT1 are mediated via the activation of eNOS, and the synergism between SIRT1 and eNOS contributes to maintaining endothelial function through positive feedback mechanisms [58]. SIRT1 binds to, deacetylates, and activates eNOS directly in an NAD-dependent manner [63]. Additionally, acetylation of eNOS inversely correlates with the activity and expression of SIRT1 [63]. Studies conducted on investigating the co-localization in the nucleus and perinuclear cytoplasm have shown that SIRT1 and eNOS are associated with each other in endothelial cells [63,64]. The interaction between SIRT1 and eNOS can accelerate the protective effect exerted against endothelial cell senescence [64]. 

Inhibition of SIRT1 decreases eNOS protein levels in both a dose- and time-dependent manner in human umbilical vein endothelial cells (HUVEC), and an endothelium-specific overexpression of SIRT1 transgenic mice leads to enhanced eNOS expression in the aorta [65]. In HUVEC, SIRT1 influences the regulation of transcriptional factors of eNOS, leading to increased eNOS protein expression and enhanced NO production [66]. In addition to eNOS expression, SIRT1 enhances eNOS enzymatic activity by performing deacetylation on the lysine 496 and 506 residues in the calmodulin-binding domain [63]. Thus, SIRT1 regulates both eNOS expression and activity. Conversely, NO derived from eNOS can also regulate SIRT1 expression. NO production exerts a protective effect against endothelial senescence and dysfunction, and upregulation of SIRT1 is modulated via an eNOS-dependent mechanism under oxidative conditions [67]. Treatment with eNOS siRNA or an eNOS inhibitor decreased SIRT1 expression and the inhibitory effect on endothelial senescence [64]. Uncoupling of eNOS decreases the expression of endothelial SIRT1, leading to endothelial progenitor cell (EPC) senescence [68]. The production of NO by calorie restriction increases SIRT1 expression, suggesting that eNOS may be involved in the regulation of SIRT1 expression in murine white adipocytes [69]. In cultured adipocytes, NO treatment markedly induced SIRT1 expression [69]. Another study using eNOS recombinant plasmid transfection showed that eNOS overexpression upregulated SIRT1 expression via direct protein-protein interaction [70]. These results demonstrate the importance of eNOS-derived NO as a regulator of SIRT1 as well as the remarkable interaction between eNOS and SIRT1, forming a positive regulatory loop in the eNOS-NO-SIRT1 axis.

## 3. CVD-Related miRNAs Target eNOS and SIRT1

Recent studies have highlighted the role of miRNA as a new powerful regulator of gene expression in endothelial cells. Studies performed on the elucidation of the link between CVD-related miRNAs and the SIRT1-eNOS/NO pathways provide new insights into the pathogenesis of vascular diseases. Thus, this chapter summarizes recent findings on nine different miRNAs, which are highly related to CVD pathology, and their target genes are involved in ROS modulation, especially focusing on the expression of eNOS, SIRT1, and antioxidant system-related enzymes (Table 1). Although eNOS and SIRT1 expression is not directly targeted, certain miRNAs act as potent regulators of redox homeostasis by targeting antioxidant system-related enzymes, such as SODs [53]. 

### 3.1. miR-155

miR-155 expression is remarkably upregulated in human atherosclerotic plaques and plasma in patients with atherosclerotic plaque rupture, indicating that miR-155 may be an essential regulator of cardiovascular function [71,72]. However, other clinical studies have shown that the level of circulating miR-155 is markedly reduced in patients with CAD compared with healthy volunteers [73,74]. Moreover, miR-155 exerts anti-angiogenic but pro-arteriogenic effects in the regulation of neovascularization after induction of hind limb ischemia in mice [94]. Additionally, miR-155 can demonstrate opposite roles in atherosclerosis development, exhibiting either pro- or anti-inflammatory properties [38].

miR-155 is a direct regulator of eNOS expression and endothelial function, and this function is realized by decreasing eNOS mRNA stability by binding to its 3′-UTR [95]. The inhibition of miR-155 expression attenuates cytokine-induced downregulation of eNOS expression and ameliorates the impairment of endothelium-dependent vasodilation [95]. In ex vivo experiments performed using mouse aortic vessels, miR-155 expression potentially inhibits vasorelaxation by eNOS-derived NO under inflammatory conditions [96]. miR-155 expression plays a role in the pathogenesis of atrial fibrillation via regulation of NO production by targeting eNOS signaling pathways [97]. Interferon-α, a potent pro-atherogenic factor, induces miR-155 expression and causes endothelial dysfunction in part through suppression of eNOS mRNA expression [98]. miR-155 is involved in the negative regulation of eNOS expression via reduction of its mRNA stability [99]. Overexpression of miR-155 in endothelial cells markedly reduces eNOS expression and NO production by directly binding to the 3′-UTR of eNOS mRNA [98]. SIRT1 is also a direct target gene of miR-155. Overexpression of miR-155 by using miR-155 mimics markedly reduces SIRT1 by directly binding to SIRT1 3′UTR in endothelial senescence in in vitro models [100]. The in vivo myocardial injury model showed consistent results, indicating that increased miR-155 expression and negatively regulated SIRT1 expression were involved [101]. In HF rats, increased expression of miR-155 and attenuated expression of SIRT1 lead to the occurrence of ventricular dysfunction [62]. 

### 3.2. miR-15/16 

Therapeutic targeting of miR-15 expression reduces infarct size and cardiac remodeling and enhances cardiac function after infliction of ischemic injury in mice experiments [75]. miR-16 expression is downregulated in coronary sinus plasma samples obtained from heart failure patients and is negatively correlated with LV filling pressure [76]. Suppression of miR-16 expression confers protection to rat heart against ischemic injury triggered by acute MI [102]. Increased miR-15/16 expression reduces endothelial cell proliferation and migration in vitro as well as capillary-like structure formation ex vivo [103]. Cardiac issues in transgenic mice overexpressing members of the miR-15 family in the heart are associated with cardiac malformation and mice show low survival owing to the impairment of cardiomyocyte proliferation and suppression of mitotic gene expression [104]. Inhibition of the expression of the members of the miR-15 family from an early postnatal age until adulthood shows higher proliferation of both cardiomyocytes and non-cardiomyocytes and improves left ventricular systolic function after adult MI [105]. miR-15 expression is upregulated in response to ischemia and contributes to the resistance of cardiac myocytes to hypoxia-induced apoptosis [75]. Inhibition of miR-15 expression remarkably suppressed the production of pro-inflammatory cytokines after infection, suggesting the protective effects of miR-15 inhibition conferred against infection-induced myocardial cell injury [106]. 

A recent report revealed a direct interaction between eNOS and miR-15 and miR-16, which induced posttranscriptional regulation of eNOS expression in endothelial cells [107]. Based on the results obtained from several studies involving animal experiments and clinical cases, inhibition of miR-15 and miR-16 expression protects against eNOS mRNA degradation and prevents the occurrence of vascular dysfunction [53]. The upregulation of miR-16 expression in the endothelium reduces eNOS expression levels and its activity, leading to impairment of endothelial recovery and NO production by repressing cell proliferation and migration [108]. In contrast, in development states, increased miR-15 and miR-16 expression regulate the function and volume of the umbilical vein, thereby providing more nutrients and oxygen from the maternal to the fetal tissue for fetal development and promoting survival by mediating the modulation of eNOS expression [109]. There is no direct evidence to prove the existence of a correlation between miR-15/16 and SIRT1 expression. A recent report showed that decreased miR-16 levels following nanocurcumin capsule treatment are associated with increased levels of SIRT1 in patients with relapsing-remitting multiple sclerosis [110]. Another member of the miR-15 family, miR-195, induces endothelial cell apoptosis by targeting SIRT1 expression, suggesting the possibility for targeting SIRT1 expression by the use of miR-15/16 [111].

### 3.3. miR-221/222 

miR-221/222 expression exerts anti-angiogenic and anti-proliferative effects by targeting c-kit expression, a pro-proliferative gene that serves as an important marker of cardiac stem cells [28,112]. miR-221/222 expression regulates essential physiological vascular processes (angiogenesis, neointimal hyperplasia, vessel wound healing, and vascular aging), and is highly involved in the regulation of vascular-related pathological mechanisms such as tumor angiogenesis, atherosclerotic inflammation, cardiac hypertrophy, and diabetic hyperglycemia-induced endothelial dysfunction [40]. Overexpression of miR-221/222 affects endothelial tube formation, indicating a potential avenue for therapeutic modulation of angiogenesis [113]. The upregulation of miR-221/222 expression markedly confers protection to cardiomyocytes against hypoxic injury via mesenchymal stem cell-mediated cardioprotection [114].

The expression levels of miR-222 increased in patients with stenosed coronary arteries following periods of cardiac stress [77]. Serum miR-221/222 levels increased remarkably in patients with carotid atherosclerosis, CAD patients, and diabetes subjects with coronary artery bypass grafts [40]. miR-222 expression is upregulated in both human HF patients and HF rats [78,79]. This upregulation of miR-221/222 expression limits the recruitment and availability of EPCs to repair vascular injury [40]. Few reports have shown that the expression of miR-221/222 is downregulated in atherosclerosis obliterans and atherosclerotic patients by influencing plaque stability [80,81].

It has also been reported that another important target mRNA of miR-221/222 is eNOS [115]. The silencing of Dicer, a key enzyme necessary for miRNA maturation, upregulated eNOS expression in the endothelial cells, and overexpression of miR-221/222 reversed elevated eNOS levels in Dicer siRNA-transfected cells via indirect mechanisms such as translational or posttranslational efficiency [115]. In placental vascular formation, miR-221/222 expression is crucial for fetal survival, growth, and development by targeting and modulating eNOS expression [109]. eNOS-suppressing miR-221/222 expression shows markedly higher levels in senescent human aortic endothelial cells, which has been associated with reduced synthesis and activity of eNOS [116]. Additionally, miR-222-3p expression directly interacts with the 3′-UTR of SOD2 in human cardiomyocytes [78]. 

### 3.4. miR-21 

The expression level of miR-21 is markedly increased in sclerotic vascular samples obtained from patients with atherosclerosis and peripheral arterial disease, plasma and endothelial cells from CAD patients, and human atherosclerotic plaques [45,81,82,83,84]. Patients with significant coronary stenosis showed an increase in circulating miR-21 levels after development of cardiac stress [77]. Additionally, the expression of miR-21 was remarkably increased in dedifferentiated VSMCs in an injured vascular wall in an in vitro model and upregulated in rat carotid arteries after infliction of balloon injury in in vivo experiments [117]. 

The functions of miR-21 in the cardiovascular system remain controversial, showing exhibition of distinct regulatory mechanisms in different cellular types [118]. Inhibition of miR-21 expression decreases cell proliferation and increases apoptosis in both cultured VSMCs and rat carotid arteries [117]. Overexpression of miR-21 promoted the proliferation of human coronary artery smooth muscle cells [82]. Increased miR-21 expression exerts protective function by inhibiting cardiomyocyte apoptosis in MI and by reducing the size of cardiomyocytes in cardiac hypertrophy, but also contributes to the pathogenesis of CVDs by leading to the development of severe fibrosis and cardiac fibroblast activation [118].

miR-21 expression directly alters SOD expression to promote ROS production. The overexpression of miR-21 increases ROS concentration and reduces hydrogen peroxide levels and NO bioavailability by targeting and suppressing the expression of SOD, whereas miR-21 inhibitors rescue endothelial function as evidenced by in vitro and ex vivo [83,119]. A recent cohort study showed the existence of an inverse association between miR-21 and SOD levels [120]. Additionally, expression of miR-21-5p and its target SOD2 has been detected in the plasma samples of HF rats [78].

Recent transcriptome profiling results indicate that miR-21 expression exerts a direct effect on eNOS expression by modulating transcript stability [121]. Another study suggested that increased miR-21 expression, induced by shear stress, contributes to the exertion of protective effects by increasing eNOS phosphorylation and NO bioavailability and by attenuating the apoptosis of endothelial cells [122]. The plasma miR-21 expression level is elevated in the carotid arteries of patients with hypertension, and miR-21 expression is negatively correlated with NO production and eNOS expression [123]. 

### 3.5. miR-199a 

The regulatory role played by miR-199a was first studied in cardiac myocytes, and its expression was rapidly reduced by exposure to hypoxia in vitro and cardiac ischemia in vivo [124]. Increased levels of miR-199a in circulating microvesicles in patients with stable CAD have prognostic values that may help reduce the risk of exhibiting major cardiovascular adverse events and revascularization in univariate analysis [44]. Additionally, miR-199a expression is upregulated in heart failure associated with diabetes [85]. The gradual decline in plasma levels of miR-199a is indicated in parallel with an increase in different manifestations of atherosclerotic disease [86]. Lower levels of miR-199a were associated with a higher risk of cardiovascular-related re-hospitalization in patients with CHF [86]. Intracardiac injection of exogenous miR-199a in mice stimulated cardiac regeneration and led to a near complete recovery of cardiac functional parameters after MI [125]. In endothelial cells, miR-199a expression has been shown to promote cell survival, proliferation, migration, and tube formation [126]. miR-199a expression plays a protective role in the cardiomyocytes of MI, and downregulation of miR-199a expression is observed in the presence of ROS [127]. Meanwhile, other recent studies suggest the opposite opinion and deny the cardioprotective function demonstrated by miR-199a. The upregulation of miR-199a expression contributes to the infliction of myocardial injury, and the downregulation of miR-199a expression can protect cardiomyocytes against infarcted cardiomyocyte apoptosis [128]. Cardiomyocyte-specific miR-199a overexpression induces the development of cardiac hypertrophy and triggers HF by impairing cardiomyocyte autophagy [129]. Inhibition of miR-199a expression promoted cardiac differentiation of embryonic stem cells, contributing to the rescue of the damaged myocardium [130]. 

SIRT1 is a direct target of miR-199a, and the downregulation of SIRT1 expression leads to increased miR-199a expression and decreased apoptosis of cardiomyocytes [124]. Moreover, the upregulation of miR-199a markedly reduced SIRT1 expression in endothelial cells, thereby enhancing angiogenic function [126]. The expression level of miR-199a is remarkably reduced and SIRT1 protein levels are markedly increased in myocardial tissues obtained from patients with CAD [131]. The oxidative stress-miR-199a-SIRT1 axis is a specific pathway for the occurrence of ischemia-driven heart failure and LV dysfunction [131].

miR-199a expression indirectly modulates eNOS expression or activity. Overexpression of miR-199a and transfection with SIRT1 siRNA resulted in a decrease in the expression of eNOS protein in endothelial cells [126]. Additionally, miR-199a induces a decrease in eNOS activation through its phosphorylation on the serine/threonine residues [132]. miR-199a expression directly targets the 3′-UTR region of SOD1 mRNA, and the blockade of miR-199a expression modulates NO bioavailability by leading to a net increase in SOD1 expression [132]. 

### 3.6. miR-34a 

miR-34a is known to regulate various target genes, including SIRT1, which controls apoptosis and cell cycle progression [133]. In endothelial cells, miR-34a also acts as a negative regulator of SIRT1 and is involved in cell senescence [134]. Overexpression of miR-34a in endothelial cells remarkably decreased SIRT1 expression and induced premature cell senescence [134]. Upregulation of miR-34a expression in mice leads to the occurrence of endothelial dysfunction by targeting SIRT1 expression, and overexpression of SIRT1 rescues miR-34a-induced endothelial dysfunction [135]. Overexpression of miR-34a induced cell death and downregulation of SIRT1 expression [88]. In a randomized clinical study, miR-34a levels were increased, but SIRT1 protein levels were lower in patients with CAD than in those without CAD [87]. 

The expression of miR-34a is highly upregulated in atherosclerotic plaques in the myocardium during CHF, and in plasma obtained from CAD patients [39,45,84]. Furthermore, miR-34a expression increased markedly in bone marrow-derived mononuclear cells isolated from patients with ischemic cardiomyopathy, acute MI (AMI), and non-ischemic heart failure [88]. Previous studies have reported that miR-34a expression inhibits EPC-mediated angiogenesis and that the number of EPCs is reduced in atherosclerotic patients, suggesting that miR-34a expression may be involved in atherosclerosis progression [84]. Inhibition of miR-34a expression improves cell survival in vitro and enhances the therapeutic benefit of cell therapy ex vivo [88]. 

### 3.7. miR-145 

miR-145, an miRNA known to be abundantly expressed in VSMCs, regulates the VSMC phenotype, induces cell proliferation and differentiation, and is involved in cardiac physiology and pathology [136]. Downregulation of miR-145 expression was first described in the injured carotid arteries of rats examined during different time courses after conducting angioplasty [117]. The expression levels of miR-145 are markedly reduced in plasma and atherosclerotic plaques from patients with CAD, and miR-145 levels are negatively associated with the disease severity [46]. Aortic miR-145 expression decreases markedly in both experimental and human atherosclerosis [89]. In humans, miR-145 levels are lower in atherosclerotic plaques than those in adjacent plaque-free regions [89]. The miR-145 level in total peripheral blood is correlated with infarct size in patients with AMI [137]. 

Overexpression of miR-145 exerts protective effects in cardiomyocytes subjected to oxidative stress in vitro [136]. Lentiviral miR-145 treatment reduces plaque size and increases atherosclerotic plaque stability in vivo [89]. miR-145 expression modulates the mitochondrial pathway to confer protection against oxidative stress-induced cardiomyocyte apoptosis as well as in the heart of mice with MI [138]. Endogenously expressed miR-145 was transiently downregulated in vivo following coronary artery occlusion in mice and in vitro upon hypoxia treatment of cardiac fibroblasts, and expression was subsequently restored [139]. miR-145 expression promotes the differentiation of cardiac fibroblasts to myofibroblasts and induces collagen expression to play a role in cardiac remodeling and scar retraction following MI [139]. 

A recent study reported that miR-145 directly targets SIRT1 expression [140]. Although no evidence related to CVDs was available thus far, one study reported that the downregulation of miR-145 expression significantly alleviated lung injury via a negative correlation between miR-145 and SIRT1, suppressing acetylation levels and transcriptional activity of pro-inflammatory cytokines [140]. Nanocurcumin capsule and resolvin D1 treatment are associated with decreased miR-145 expression and increased levels of SIRT1 in multiple sclerosis and uveitis, suggesting that miR-145 may be a key regulator of SIRT1 [110,141]. Further studies are warranted to obtain more precise information regarding the relationship between miR-145 and SIRT1 as well as their function in multiple pathological conditions, including CVDs.

### 3.8. miR-210 

miR-210 is a well-known master miRNA involved in the elicitation of hypoxic responses because its expression is upregulated by hypoxia in all the cell types analyzed thus far [4,42]. The expression of miR-210 in endothelial cells is increased under hypoxia conditions [85]. The levels of miR-210 are elevated in the plasma of patients with heart failure, human myocardium of infarcted human hearts, vascular plaque samples obtained from patients with atherosclerosis, and sclerotic samples and serum samples obtained from patients with arteriosclerosis obliterans [41,45,81,90,91]. Upregulation of miR-210 expression is also involved in the progression of atherosclerosis both in vitro and in vivo [41,142]. 

However, controversial data are reported on the exertion of miR-210 effects on ROS production and cardioprotective function. Overexpression of miR-210 achieved by transfection contributed to functional recovery of the ischemic heart and stem cell-based exogenous miR-210 delivery to the infarcted heart would be an effective strategy to preserve LV function [143]. Intracardiac injections of miR-210 in a mouse model of MI promoted improvement of LV fractional shortening, decreased apoptosis of cardiomyocytes, and increased neovascularization and cardiac function [144] In contrast, the upregulation of miR-210 expression was associated with increased endothelial apoptosis [142]. The inhibition of miR-210 expression improved survival and cardiac function after development of AMI in vivo [91].

miR-210 directly targets iron/sulfur cluster assembly enzymes (ISCU) 1 and 2 isoforms, causing electron leakage and increased levels of ROS via superoxide anion production [145]. Additionally, an opposite trend has been reported on the effects of miR-210 on ROS. In cardiomyocytes, miR-210 expression is upregulated and the overexpression of miR-210 reduces mitochondrial ROS production [146]. miR-210 regulates SIRT3 expression indirectly through the regulation of ISCU, suggesting links between the sirtuin family and miR-210 under oxidative stress [147]. Moreover, recent evidence has shown that miR-210 directly targets the SIRT1 gene to increase acetylation of NF-κB p65 binding and activate NF-κB signaling, thereby triggering microglial-mediated neuroinflammation [148]. This report provides new evidence for the direct association between miR-210 and SIRT1. Future studies are warranted to determine whether miR-210 targeting SIRT1 contributes to endothelial function and to ascertain its association with the development of CVDs. 

### 3.9. miR-126 

A study by using genetic deletion of miR-126 in mice revealed that an essential role for miR-126 in angiogenesis and maintenance of vascular integrity [149]. Inhibition of miR-126 expression impairs endothelial outgrowth ex vivo and ischemia-induced angiogenesis in vivo [150]. miR-126 expression regulates vascular inflammation by inhibiting the expression of vascular cell adhesion molecule 1 (VCAM-1), which mediates leukocyte adherence to endothelial cells [151]. The upregulation of miR-126 expression promotes myocardial angiogenesis by suppressing negative regulators of the VEGF pathway [93]. miR-126 expression is enriched in endothelial cell-derived apoptotic bodies from atherosclerotic plaques, reduces inflammatory cytokine-mediated plaque development, and consequently conducts vascular protection [152]. A higher level of miR-126 expression in isolated microvesicles is associated with a reduced risk of developing major cardiovascular adverse events in patients with stable CAD [44]. 

Plasma concentration of miR-126 is negatively correlated with the severity of heart failure [153]. Circulating miR-126 expression is significantly downregulated in the plasma of patients with acute MI and CAD [74,92]. Furthermore, in circulating microvesicles obtained from patients with stable CAD, the miR-126 expression is remarkably reduced compared with healthy subjects [44]. Low expression of miR-126 is associated with an increased risk of cardiovascular disease-associated death in patients with chronic heart failure caused by ischemic cardiomyopathy and non-ischemic heart failure [93]. 

Overexpression of miR-126 inhibited ROS levels and increased SOD expression in EPCs under oxidative stress conditions [154]. These endothelial cell damage-relieving functions of miR-126 are mediated by decreasing SOD expression and activation of the eNOS signaling pathway [155]. SIRT1 may also be involved in miR-126 regulation. The downregulation of miR-126 is correlated with SIRT1 downregulation under oxidative stress [59]. Aberrant miR-126 expression indirectly represses SIRT1 expression by deregulating p53, a bona fide substrate of SIRT1 deacetylation [156]. 

## 4. The Regulation of CVD-Related miRNAs by Natural Antioxidants

Since it has been established that oxidative stress plays an important role in cardiovascular pathologies, several studies have been conducted to investigate the therapeutic effects of antioxidant therapy. Many studies have demonstrated the beneficial effects of natural antioxidants against CVDs, including atherosclerosis, hypertension, ischemia/reperfusion, and heart failure to diabetes, obesity, and aging. This protective effect is supported by results of several studies conducted in both animal models and human clinical studies with isolated flavonoids and flavonoid-rich food consumption, respectively [23]. The action of antioxidant molecules is mediated via interactions with multiple cell-signaling pathways by modulating the activity of transcription factors and gene expression [27]. Accumulating evidence suggests that the modulation of miRNAs may be an important biological strategy to attenuate pathological conditions of CVDs by natural antioxidants derived from dietary supplementation [157]. In this chapter, we have discussed the regulatory networks, specifically related to the SIRT1/eNOS signaling pathway, mediated by several natural antioxidants via expression of CVD-related miRNAs, which have already been discussed in Chapter 3 (Table 2). 

### 4.1. Stilbenes

Stilbenes are natural phenolic defense compounds that act as antifungal phytoalexins in response to infection or injury in many plant species [26]. Resveratrol (3,5,4-trihydroxy-trans-stilbene) is a well-known polyphenol phytoalexin that is found mainly in the skin of grapes [192]. Resveratrol can directly activate SIRT1 and reduce endothelial oxidative stress by promoting NO production, by upregulating eNOS expression, by increasing eNOS activity, by preventing eNOS uncoupling, and by enhancing antioxidant enzymes in endothelial cells [192]. Knockdown of SIRT1 by siRNA completely inhibited the decrease in ROS levels by resveratrol, indicating that the antioxidative function of resveratrol depended on SIRT1 expression in cardiomyocytes [193]. Treatment with resveratrol increased SIRT1 expression, eNOS expression, and eNOS activation, and inhibited a senescent endothelial phenotype [64]. 

Resveratrol is one of the most studied polyphenols with a well-established cardioprotective role. The SIRT1-dependent cell protective functions of resveratrol suppressed the formation of fibrosis, preserved cardiac function, and markedly improved survival [193]. Resveratrol induced cardioprotection against MI in rat hearts and improved diabetes-induced vascular dysfunction by increasing the expression and activation of eNOS and by enhancing endothelial cell healing through SIRT1 expression [194,195]. Administration of resveratrol increased the mRNA and protein levels of SOD via SIRT1 activation in cardiomyocytes [193]. 

Under tumor necrosis factor (TNF)-α-induced inflammatory conditions, resveratrol attenuates endothelial inflammation and induces SIRT1 expression in endothelial cells [169]. The expression of miR-221/222 is suppressed in TNF-α-treated endothelial cells, which is reversed by resveratrol treatment [169]. Resveratrol inhibits endothelial cell apoptosis by upregulating miR-126 expression [189]. 

Other miRNA expression levels are downregulated by resveratrol treatment. Human cytomegalovirus infection upregulates miR-199a expression in endothelial cells, and cell migration and tube formation are promoted via the downregulation of SIRT1/eNOS expression [126]. Pretreatment with resveratrol downregulates miR-199a levels, inhibits motility and tube formation in infected endothelial cells, and this effect is reversed by the application of SIRT1 siRNA [126]. 

Low doses of resveratrol increase re-endothelialization ex vivo and reduce neointima formation in vivo after infliction of endothelial injury [196]. This role of resveratrol is realized by inhibiting miR-21 expression in endothelial stem/progenitor cells, leading to endothelial differentiation [170]. Treatment using resveratrol and its commercial formulation, longevinex, results in considerable downregulation of miR-21 expression and cardioprotection against ischemia/reperfusion injury [157]. These results suggest that miR-21 expression is directly associated with resveratrol usage in cardiovascular diseases both in vitro and in vivo. 

The suppressive effect of resveratrol on miR-34a expression upregulation plays an important role in SIRT1 restoration in cardiomyocytes after cardiac injury inflicted by ischemia-reperfusion [197]. Resveratrol reduces the expression of cardiac inducible NOS and miR-34a in a rat model of myocardial injury [182]. Both miR-34a overexpression and SIRT1 knockdown markedly decreased the effect of resveratrol on the reduction of ROS generation and improvement of the viability of cardiomyocytes [197]. Resveratrol also decreased the levels of miR-34a in a myocardial fibrosis in vitro model along with the usage of transforming growth factor-β1 (TGF-β1), the most influential profibrogenic factor [183].

Administration of resveratrol decreases the expression of miR-155 in a dose-dependent manner in vitro and attenuates cardiac myocyte hypertrophy both in vitro and in vivo [158]. Resveratrol impairs the LPS-induced upregulation of miR-155 expression, a hallmark miRNA of the innate immune response, in human peripheral blood monocytes [159]. Inhibition of miR-155 expression by resveratrol treatment is detected in LPS-treated macrophages, ischemic brain, and adipocytes [198,199,200]. These results suggest that resveratrol modulates miR-155 expression to reduce the inflammatory response and to confer protection against diabetes, atherosclerosis, and hypertension [160,200]. One year of supplementation of resveratrol-enriched grape extract in hypertensive patients with CAD downregulated the expression of pro-inflammatory cytokines via regulation of the expression levels of miR-21, miR-155, and miR-34a [160]. 

Additionally, pterostilbene, a dimethylated resveratrol analog, reduces cardiac ROS production and alleviates myocardial injury by increasing miR-15b expression in cardiomyocytes [165]. However, the effects of stilbenes on miR-145 and miR-210 remain unknown in endothelial cells and/or cardiovascular diseases. 

### 4.2. Other Non-Flavonoid Polyphenols

Phenolic compounds account for approximately 30% of the polyphenols reported and are found in all plant materials, and the compounds are particularly abundant in sour-tasting fruits in various forms, including gallic acid, caffeic acid, and ferulic acid [26]. Gallic acid (3,4,5-trihydroxybenzoic acid), the precursor of multiple plant-derived tannins, is one of the most studied and promising compounds among the phenolic acids investigated [26]. In nature, gallic acid and its derivatives are abundant in strawberries, bananas, pineapples, gallnuts, oak bark, wood, leaf, and other plants [26]. *Nelumbo nucifera* leaf polyphenol extract and its main component, gallic acid, inhibited VSMC proliferation and migration to decelerate atherosclerosis progression by downregulating miR-21 expression and by upregulating miR-145 expression [172]. Garlic is a natural component enriched in phenolic acid that provides considerable health benefits including the prevention and treatment of several disorders such as CVDs [201]. Garlic treatment increased the expression of miR-126 and miR-210 in rat myocardial tissue, enhanced cardiac angiogenesis, and improved serum lipid profile [42].

Extra virgin olive oil (EVOO) is a cornerstone of the Mediterranean diet and demonstrates health-promoting effects such as a lower incidence of CVDs [202]. Hydroxytyrosol, a major phenolic alcohol present in EVOO, functions as a scavenger of ROS and possesses antioxidant properties [173]. The combination of hydroxytyrosol and NO increased the phosphorylation of eNOS through SIRT1 induction in the thoracic aorta of mice and HUVECs [203]. Highly-enriched EVOO consumption decreased circulating miR-21-5p levels in plasma obtained from healthy volunteers [173]. Another study using a rat cardiovascular injury model subjected to ozone exposure showed that EVOO enhanced cardiac function, and its beneficial effect was associated with the upregulation of miR-21 expression [171]. 

Salidroside is isolated from *Rhodiola rosea*, which belongs to a group of plant phenolic compounds called phenylethanoids, and is used as an herbal medicine exhibiting pharmacological properties, including antioxidative and cardioprotective properties [204]. According to a recent study, enhancement of the miR-21 expression results in salidroside-mediated protective effects against myocardial oxidative stress and inflammatory response [205]. 

Curcuminoids, especially curcumin, are natural polyphenols derived from the roots of *Curcuma longa*, which is responsible for the yellow color of turmeric [190]. Extensive studies have demonstrated that curcumin has a variety of therapeutic activities owing to its antioxidant properties. However, several mechanistic studies related to miRNA regulation by curcumin are limited in cancer research. According to a recent study, curcumin also exerts potent anti-atherosclerosis effects by upregulating miR-126 expression [206].

### 4.3. Flavonoids

The most well-known and researched flavonols are kaempferol and quercetin. Kaempferol reduces the formation of cardiac fibrosis and cellular apoptosis in diabetic cardiomyopathy by repressing oxidative stress and inflammation [207]. Furthermore, kaempferol treatment can improve cardiac function and suppress MI via a decrease in myocardial infarct size, cardiomyocyte apoptosis, oxidative stress, and inflammatory responses [208]. Kaempferol exhibits protective roles against myocardial cell injury model by decreasing the expression of miR-15b [167]. Kaempferol inhibited VSMC proliferation and migration by inducing miR-21 expression, thereby showing preventative effects against cardiovascular diseases relevant to VSMC migration disorders [174]. Enhancement of the miR-21 expression also mediates kaempferol-induced cardioprotection in cardiomyocytes against myocardial injury by reducing oxidative stress [209]. Transfection of the miR-21 inhibitor eliminates the protective effect of kaempferol exerted on oxidative stress by reversing the enhanced activities of antioxidant enzymes such as SOD and attenuates iNOS activity and NO levels [209]. Quercetin is the most common and widely distributed flavonol compound, and daily consumption is realized in the form of plant-derived foods such as tea, onion, lettuce, broccoli, beans, and buckwheat [210]. Quercetin has a long history of exerting beneficial health effects, especially cardioprotective effects, and is associated with the ability to quench free radicals as antioxidants and to reduce the formation of atherosclerotic plaque [210]. The anti-inflammatory properties of quercetin include the downregulation of miR-155 enhancement of the miR-21 expression, which is one of the pro-inflammatory miRNAs [161]. Quercetin mitigates cell viability reduction and ameliorates miR-199a accumulation in cardiomyocytes under hypoxia [181]. Decreasing miR-199a expression by using quercetin is implicated in SIRT1 expression upregulation by directly targeting its 3′-UTR [181]. 

Among the flavonoids, flavanols have been identified as the bioactive phytochemicals present in green tea and the major members of flavanols include epicatechin, epigallocatechin, and epicatechin gallate (EGCG) [211]. EGCG is the most studied flavanol molecule conjugated with gallic acid [27]. Among catechins, EGCG exhibits the most remarkable antioxidant activity, and is a key molecule that contributes to the potential health benefits of green tea consumption [26]. Green tea consumption is correlated with a low incidence of chronic CVDs [27]. EGCG plays a major role in cardiovascular health-promoting activities, including anti-atherosclerosis, anti-cardiac hypertrophy, anti-myocardial infarction, anti-inflammatory, and antioxidant activities [212]. EGCG consumption reduces oxidative stress and enhances the restoration of cardiac function against cardiomyopathy [213]. Another recent report showed that the cardioprotective effect of EGCG could inhibit pressure overload-induced cardiac hypertrophy via the SIRT6-related signaling pathway [214]. miR-145 levels decreased in LV myocardium in MI rats, and treatment with EGCG markedly reversed the expression [186]. 

Flavanones and flavones are mainly found in high concentrations in the same citrus fruits, such as oranges and lemons [17]. Apigenin, a major plant flavone present in chamomile, can alleviate MI after AMI [168]. Apigenin plays a role in conferring protection against myocardial injury by reducing the expression of miR-15b [168]. Apigenin also attenuates TGF-β1-stimulated cardiac fibroblast differentiation, and its mechanisms are associated with the reduction in miR-155-5p expression [162]. Luteolin is another major flavone, mainly present in carrots, artichoke, basil, celery, and parsley, possessing antioxidant activities and cardioprotective properties [176]. Luteolin treatment suppresses oxidative stress and cardiac fibrosis, and this effect is highly associated with the reduction in miR-21 expression in an in vivo model of myocardial injury [176]. The flavone baicalin is a major component isolated from the root of the medicinal herb, *Scutellaria baicalensis Georgi*, and has extensive pharmacological properties that can be considered for the treatment of CVDs [215]. The expression of miR-126-5p was reduced in the peripheral blood of patients with atherosclerosis and oxidized low-density lipoprotein-treated VSMCs (ox-LDL-VSMCs), and baicalin treatment inhibited ox-LDL-VSMC proliferation and migration by upregulating miR-126-5p expression [191]. 

Dihydromyricetin, also known as ampelopsin, is a flavanonol with anti-inflammatory, antioxidant, and anti-tumor properties [216]. Dihydromyricetin alleviates TNF-α-induced endothelial dysfunction via miR-21 expression downregulation, resulting in enhanced eNOS phosphorylation and NO production in vitro [177]. A recent report shows a consistent result that dihydromyricetin decreases miR-21 expression, improves endothelial function, resulting in the inhibition of vascular inflammation and plaque formation in in vivo models of atherosclerosis [178]. Dihydromyricetin is also highly associated with the miR-155 and miR-34a-mediated autophagy signaling pathways, but there is no information available on the direct regulation of miR-155 and miR-34a in endothelial dysfunction and CVDs [216,217].

Puerarin, an isoflavone derived from the root of the kudzu plant, has remarkable biological activities as a traditional Chinese medicine used for the treatment of CVDs, including myocardial injury [175]. Puerarin enhances endogenous miR-21 expression, which has a significant role in the exertion of cardioprotective effects in an in vitro model of myocardial injury [175]. Puerarin exerts strong effects by attenuating cardiac hypertrophy through the promotion of miR-15b expression in a mouse cardiac hypertrophy model and primary cardiomyocytes [166]. Another isoflavone compound, genistein, is the most abundant in soy and soy products and plays an important role in the prevention of atherosclerosis [164]. Genistein decreases the expression of miR-34a and miR-155 in endothelial cells under conditions of oxidative damage and inflammation [163,164]. Inhibition of miR-34a expression by genistein is associated with a reduction in ROS production and an inhibitory effect exerted on SOD and CAT by the upregulation of SIRT1 expression [163]. 

### 4.4. Terpenoids

Crocin is a carotenoid that is responsible for the characteristic color of saffron and possesses a variety of pharmacological properties such as antioxidant effects, leading to the neutralization of free radicals [218]. Crocin administration exerts cardioprotective effects against inflammation and oxidative stress [219]. Crocin exerts a protective effect by decreasing MI size and by enhancing the expression of genes responsible for antioxidant enzymes to restore the balance between iNOS and eNOS levels in the myocardium of MI hearts [220]. A recent study has reported that miR-34a expression is altered in the myocardium after myocardial injury and overexpression of miR-34a aggravates myocardial injury by increasing infarct size and by decreasing LV function [221]. This miR-34a functions via negative regulation of SIRT1, a potent target of miR-34a [221]. Crocin exerts cardioprotective effects against myocardial injury by decreasing cardiomyocyte apoptosis, by suppressing miR-34a upregulation after injury of cardiomyocytes, and by enhancing SIRT1 expression [184]. In contrast, crocin treatment increases miR-126 and miR-210 expression in rat cardiac tissue [188]. Increased miR-126 and miR-210 expression induced cardiac capillary formation and improved cardiac angiogenesis [188]. 

Geniposide, a major iridoid compound extracted from the gardenia fruit, *Gardenia jasminoides* (Rubiaceae), is a traditional Chinese medicine with a broad spectrum of pharmacological activities, such as conferring protection against inflammatory injury and stress [222]. Recent studies have focused on the potential cardioprotective effects of geniposide, demonstrating that geniposide treatment reduces plaque size and alleviates atherosclerosis-associated inflammatory injury [223]. Geniposide attenuates endothelial injury and ROS generation by decreasing antioxidant enzyme activities, including SOD and CAT, which are directly associated with the enhancement of miR-21 expression in animal models of atherosclerosis [222]. The protective effect of geniposide on injured cardiomyocytes is also related to the upregulation of miR-145 expression [187].

Ursolic acid is a natural terpene compound found in various medical herbs and fruits, such as apple peel, cranberries, rosemary, lavender, peppermint, and thyme, exhibiting many pharmaceutical properties. Ursolic acid attenuates cardiac hypertrophy and myocardial fibrosis in vivo by inhibiting the expression of miR-21 [180].

Panax notoginseng saponins are one of the phytosterol compounds derived from the roots of Panax notoginseng, traditionally used as a hemostatic medicine in China for thousands of years. Increasing evidence has focused on their use for the treatment of CVDs owing to their vasodilatory and antihypertensive properties [224]. Notoginsenoside, a major component of Panax notoginseng, exerts cardioprotective effects via upregulation of miR-21 expression [179]. Another effective saponin, astragaloside, protects cardiomyocytes by reducing excessive oxidative stress, by decreasing the expression of miR-34a, and by promoting SIRT1 expression [185]. 

## 5. Conclusions and Future Perspectives

Physiological ROS production is essential for the maintenance of vascular homeostasis, but excessive ROS production increases oxidative stress, resulting in impaired endothelial function and manifestation of the adverse effects of CVDs [2,225]. Under normal physiological conditions, ROS generation is intricately regulated via antioxidant defense mechanisms involved in scavenging endogenous ROS such as SODs and CATs [53]. However, under pathophysiological conditions, this antioxidant system is impaired and induces oxidative stress by increasing the levels of pro-oxidant species [53]. Endothelial dysfunction is defined as a reduction in the bioavailability of vasodilators, such as NO, whereas levels of vasoconstrictors, such as ROS, are increased [52]. Since this imbalance between a vasoconstrictor and vasodilatory marker is a notable feature of the overall cardiovascular risk factor burden, endothelial dysfunction is regarded as the “ultimate risk of the risk factors” [3]. Endothelial dysfunction has been proposed to possess pathophysiological importance in the atherosclerotic process and is a high risk for the occurrence of cardiovascular events in patients with hypertension or diabetes [52]. 

Oxidative stress leads to eNOS dysfunction, and the production and bioactivity of NO are inhibited. Although several studies have reported the transcriptional and posttranslational regulation of eNOS, such as the activity of the eNOS promoter, changes in eNOS mRNA expression and interactions with other proteins, the regulation mechanisms of eNOS remain incompletely understood and other novel regulators should be discovered [226]. SIRT1 expression contributes to a key mechanism in the pathology of many diseases, including CVDs [227]. In endothelial cells, SIRT1 activation alleviates the oxidative stress response and promotes eNOS-derived NO bioavailability [4]. Endothelial SIRT1 expression exerts the function of vasoprotection by preventing endothelial dysfunction and cell senescence by promoting endothelial angiogenesis and migration and by suppressing vascular inflammation and macrophage foam cell formation [228]. The upregulation and activation of SIRT1 expression confer cellular protection against oxidative stress in the vascular system, whereas decreased SIRT1 expression by excessive ROS production or aging causes the occurrence of endothelial dysfunction [4]. However, investigation of the specific molecular mechanisms that modulate SIRT1 and eNOS interaction in oxidative stress-induced endothelial dysfunction is vital. 

The integrative data presented here strengthen the concept that miRNAs constitute an important target for a therapeutic strategy against CVDs by targeting ROS regulation-related genes that are highly associated with the occurrence of endothelial dysfunction, especially those focused on SIRT1 and eNOS expression (Table 1). Furthermore, since the cardioprotective abilities of dietary nutrition are linked to the regulation of gene expression, it is important to understand the mechanism by which natural antioxidant-mediated cardiac gene expression is controlled at the level of transcriptional regulation [157]. Therefore, the discovery of new miRNAs can enhance our understanding of cardiovascular function-associated gene expression at the posttranscriptional level. The evidence mentioned above indicates that miRNAs play an important role in regulating oxidative stress responses in CVDs, and natural antioxidant consumption can contribute to the exertion of cardioprotective effects by modulating the expression of these miRNAs (Table 2). 

We summarized the functions of nine different miRNAs, including three cardioprotective miRNAs (miR-221/222, miR-145, and miR-126), two cardiopathogenic miRNAs (miR-155 and miR-34a), and four miRNAs with unknown precise function in CVD pathology (miR-15/16, miR-21, miR-199a, and miR-210). Expressional changes in these miRNAs mediated by the consumption of four different subgroups of natural antioxidants were reviewed. Stilbenes, mainly resveratrol, induced upregulation of cardioprotective miR-221/222 and miR-126 expression, and downregulation of cardiopathogenic miR-155 and miR-34a expression. The results indicate that the upregulation of miR-15/16 expression and the downregulation of miR-21 and miR-199a expression can be considered to speculate their functional direction despite persisting controversies. However, in the case of miR-15/16 and miR-21, two opposite effects were observed which were mediated by other natural antioxidants, and this insufficient and discordant information warrants further exploration in the future. As almost all miRNA studies and/or natural antioxidant studies have focused on therapy of cancer rather than that of CVDs, further evidence is necessary to support the therapeutic potential of miRNAs in CVDs in the future. Several miRNAs showed inconsistent results in their expression changes in CVD patients depending on different types of CVDs present in the patients or sample types collected from patients (Table 1). The relationship between natural antioxidants and miRNAs remains elusive (Table 2). Nevertheless, insights into the understanding of these molecular mechanisms and the discovery of novel miRNAs and their target genes and related endothelial dysfunctions are of paramount importance.

## Figures and Tables

**Table 1:** miRNA expression in CVD patients, their function in the pathology of CVDs, and their potential target genes involved in ROS level regulation.

	Expression Levels in CVD Patients	Function in CVDs	Target Genes
miR-155	Up [71,72] Down [73,74]	Pathogenic	eNOS SIRT1
miR-15/16	Up [75] Down [76]	Controversial	eNOS SIRT1 (possible)
miR-221/222	Up [40,77,78,79] Down [80,81]	Protective	eNOS SOD2
miR-21	Up [45,77,81,82,83,84]	Controversial	eNOS SOD2
miR-199a	Up [44,85] Down [86]	Controversial	SIRT1 SOD1 eNOS (indirectly)
miR-34a	Up [39,45,84,87,88]	Pathogenic	SIRT1
miR-145	Down [46,89]	Protective	SIRT1
miR-210	Up [41,45,81,90,91]	Controversial	SIRT1
miR-126	Down [44,74,92,93]	Protective	SOD SIRT1 (possible)

**Table 2:** Changes in the expression of miRNAs in CVDs by treatment with natural antioxidants.

	Stilbenes (Resveratrol)	Other Non-Flavonoids Polyphenols	Flavonoids	Terpenoids
miR-155	Down [158,159,160]	–	Down Quercetin, Apigenin, Genistein [161,162,163,164]	–
miR-15/16	Up Pterostilbene [165]	–	Up Puerarin [166] Down Kaempferol, Apigenin [167,168]	–
miR-221/222	Up [169]	–	–	–
miR-21	Down [157,160,170]	Up EVOO, Salidroside [171] Down Gallic acid, Hydroxytyrosol [172,173]	Up Kaempferol, Puerarin [174,175] Down Luteolin, Ampelopsin [176,177,178]	Up Saponin [179] Down Ursolic acid [180]
miR-199a	Down [126]	–	Down Quercetin [181]	–
miR-34a	Down [157,160,182,183]	–	Down Genistein [163,164]	Down Crocin, Saponin [184,185]
miR-145	–	Up Gallic acid [172]	Up EGCG [186]	Up Geniposide [187]
miR-210	–	Up Garlic [42]	–	Up Crocin [188]
miR-126	Up [189]	Up Garlic, Curcumin [42,190]	Up Baicalin [191]	Up Crocin [188]

## Data Availability

Data sharing not applicable. No new data were created or analyzed in this study. Data sharing is not applicable to this article.
